# Idiopathic Intracranial Hypertension with and Without Papilledema: A Comparative Cohort Study

**DOI:** 10.3390/brainsci16080798

**Published:** 2026-07-28

**Authors:** Hafize Nalan Güneş, Burcu Gökçe Çokal, Melih Altıkardeş, Gözde Orman, Sevim Kuyumcu, Gürsel Güneş, Levent Ertuğrul İnan

**Affiliations:** 1 Department of Neurology, Ankara Training and Research Hospital, University of Health Sciences, Ankara 06018, Türkiye; gokceburcu@gmail.com (B.G.Ç.); altikardesmelih6@gmail.com (M.A.); drleinan@yahoo.com (L.E.İ.); 2 Department of Ophthalmology and Neuro-Ophthalmology, Ankara Training and Research Hospital, University of Health Sciences, Ankara 06018, Türkiye; gozdeerkan@hotmail.com; 3 Department of Ophthalmology, Acıbadem Kozyatağı Hospital, Istanbul 34734, Türkiye; sevimkavuncu@yahoo.co.uk; 4 Department of Internal Medicine and Hematology, Ankara Etlik City Hospital, Ankara 06170, Türkiye; gursel_gunes@hotmail.com

**Keywords:** headache, idiopathic intracranial hypertension, idiopathic intracranial hypertension without papilledema, papilledema, visual field defects

## Abstract

**Highlights:**

**What are the main findings?**

- Nearly one-third of patients with idiopathic intracranial hypertension (IIH) were retrospectively classified as having idiopathic intracranial hypertension without papilledema (IIHWOP) based on the available clinical, lumbar puncture, and neuroimaging findings.
- Exploratory analyses suggested that patients with IIHWOP may have a higher frequency of migraine-associated symptoms, including photophobia, phonophobia, and nausea/vomiting, compared with patients with papilledema. The observed difference in optic nerve flattening should be interpreted within the context of the diagnostic framework used to classify IIHWOP.

**What are the implications of the main findings?**

- The absence of papilledema should not, by itself, exclude the possibility of clinically significant intracranial hypertension in appropriately selected patients with supportive clinical, lumbar puncture, and neuroimaging findings.
- Comprehensive neuro-ophthalmologic and radiological evaluation may assist the diagnostic assessment of suspected IIH when papilledema is absent.

**Abstract:**

**Background:** Idiopathic intracranial hypertension without papilledema (IIHWOP) is increasingly recognized, but its clinical characteristics and treatment-related outcomes remain insufficiently defined. This study aimed to compare patients with idiopathic intracranial hypertension (IIH) with and without papilledema. **Methods:** This retrospective observational cohort study included adult patients diagnosed with IIH at a tertiary neurology and referral center. Demographic, clinical, neuro-ophthalmologic, radiological, lumbar puncture, and treatment-related characteristics were compared according to papilledema status. **Results:** Among 123 patients with IIH, 83 (67.5%) had papilledema, and 40 (32.5%) had IIHWOP. Age, sex distribution, body mass index, lumbar puncture opening pressure, and most neuroimaging findings were similar between groups. In exploratory between-group analyses, photophobia [35/40 (87.5%) vs. 55/83 (66.3%), *p* = 0.023], phonophobia [35/40 (87.5%) vs. 56/83 (67.5%), *p* = 0.031], and nausea/vomiting [20/40 (50.0%) vs. 17/83 (20.5%), *p* = 0.002] were more frequent among patients with IIHWOP than among those with papilledema. Diabetes mellitus was also more frequent in the IIHWOP group [8/40 (20.0%) vs. 5/83 (6.0%), *p* = 0.040]. Treatment persistence did not differ significantly between groups (log-rank *p* = 0.604). In multivariable analysis, papilledema was associated with higher odds of failure to achieve a complete treatment response, whereas higher lumbar puncture opening pressure was associated with lower odds of treatment nonresponse. **Conclusions:** Exploratory analyses suggested that patients with IIHWOP may exhibit a different clinical presentation characterized by more prominent migraine-like symptoms. However, these exploratory observations require confirmation in prospective studies.

## 1. Introduction

Idiopathic intracranial hypertension (IIH) is a neurological disorder characterized by elevated intracranial pressure (ICP) in the absence of an intracranial mass lesion, hydrocephalus, infection, or cerebral venous thrombosis [1]. The condition predominantly affects women of reproductive age and is strongly associated with obesity [2]. Although its pathophysiology remains incompletely understood, impaired cerebrospinal fluid (CSF) dynamics, venous sinus abnormalities, metabolic factors, and obesity-related mechanisms are believed to contribute to disease development [1,3]. Recent evidence further supports IIH as a multifactorial disorder involving metabolic, hormonal, venous, and CSF regulatory pathways [4,5]. Metabolomic studies have identified distinct systemic metabolic alterations in newly diagnosed patients, reinforcing the role of metabolic dysregulation in disease pathogenesis [6]. Clinically, IIH is important because it may cause permanent visual impairment while imposing a substantial burden of chronic headache and reduced quality of life.

Papilledema is traditionally regarded as the hallmark ophthalmologic manifestation of IIH and remains a key component of contemporary diagnostic frameworks [1,7]. Consequently, most clinical studies and management strategies have focused on patients with visible optic disc edema. Current consensus guidelines emphasize preservation of visual function, reduction in intracranial pressure, and management of headache-related disability as the primary therapeutic goals [1].

However, increasing evidence indicates that elevated intracranial pressure may occur in the absence of papilledema, a condition known as idiopathic intracranial hypertension without papilledema (IIHWOP) [8,9]. Although initially considered uncommon, recognition of IIHWOP has increased over the past decade. Patients frequently present with chronic headache syndromes, migraine-like symptoms, visual complaints, and radiological signs of raised intracranial pressure despite lacking the classic funduscopic finding of optic disc swelling [10,11]. As a result, diagnosis may be delayed or overlooked, particularly when neuro-ophthalmologic abnormalities are subtle [9,11,12].

The diagnostic evaluation of IIHWOP remains challenging [12]. Current diagnostic frameworks may lack sufficient sensitivity for identifying papilledema-negative disease, potentially leading to underrecognition of patients with clinically significant intracranial hypertension despite supportive clinical and radiological findings [9,10,11]. Neuroimaging markers such as empty sella, optic nerve sheath distention, posterior globe flattening, and transverse venous sinus stenosis have emerged as important adjuncts in supporting the diagnosis when papilledema is absent [13,14,15]. Furthermore, accumulating evidence suggests that headache burden, visual complaints, and visual field abnormalities may occur even without papilledema, indicating that optic disc edema alone may not fully reflect the clinical spectrum or disease burden of IIH [9,16].

Despite growing awareness of IIHWOP, direct comparisons between patients with and without papilledema remain limited. Most available studies consist of small case series, highly selected populations, or investigations focused primarily on diagnostic criteria rather than comprehensive clinical characterization [9,10,16]. Consequently, important questions remain regarding whether papilledema status is associated with differences in symptom profile, neuro-ophthalmologic findings, imaging features, lumbar puncture (LP) parameters, treatment patterns, and clinical outcomes.

A better understanding of these differences may improve diagnostic recognition, facilitate earlier intervention, and refine current approaches to risk stratification and management. Therefore, the present study compared demographic, clinical, neuro-ophthalmologic, radiological, and LP characteristics between patients with IIH with and without papilledema. In addition, we evaluated treatment persistence and predictors of complete treatment response to determine whether papilledema status was associated with differences in therapeutic outcomes.

## 2. Materials and Methods

### 2.1. Study Design and Participants

This retrospective observational cohort study included adult patients diagnosed with IIH at a tertiary neurology and neuro-ophthalmology referral center. The medical records of consecutive adult patients diagnosed with IIH between January 2015 and December 2024 were retrospectively screened for eligibility. The study was approved by the local Institutional Review Board (approval no. E-26-970; 25 June 2026) and was conducted in accordance with the Declaration of Helsinki. Written informed consent was obtained from all participants.

IIH was diagnosed according to the modified Dandy criteria and contemporary consensus recommendations, requiring symptoms and signs of elevated intracranial pressure, elevated CSF opening pressure with normal CSF composition, exclusion of secondary causes on neuroimaging, and exclusion of alternative causes of intracranial hypertension [17]. Patients without papilledema were retrospectively classified as having IIHWOP using the revised Friedman framework [17], based on elevated LP opening pressure, normal CSF composition, exclusion of secondary causes, and supportive neuroimaging findings documented in routine clinical records. The available imaging variables included empty or partially empty sella, posterior globe flattening, documented optic nerve-related abnormalities, and MR venographic findings.

**Figure 1:**
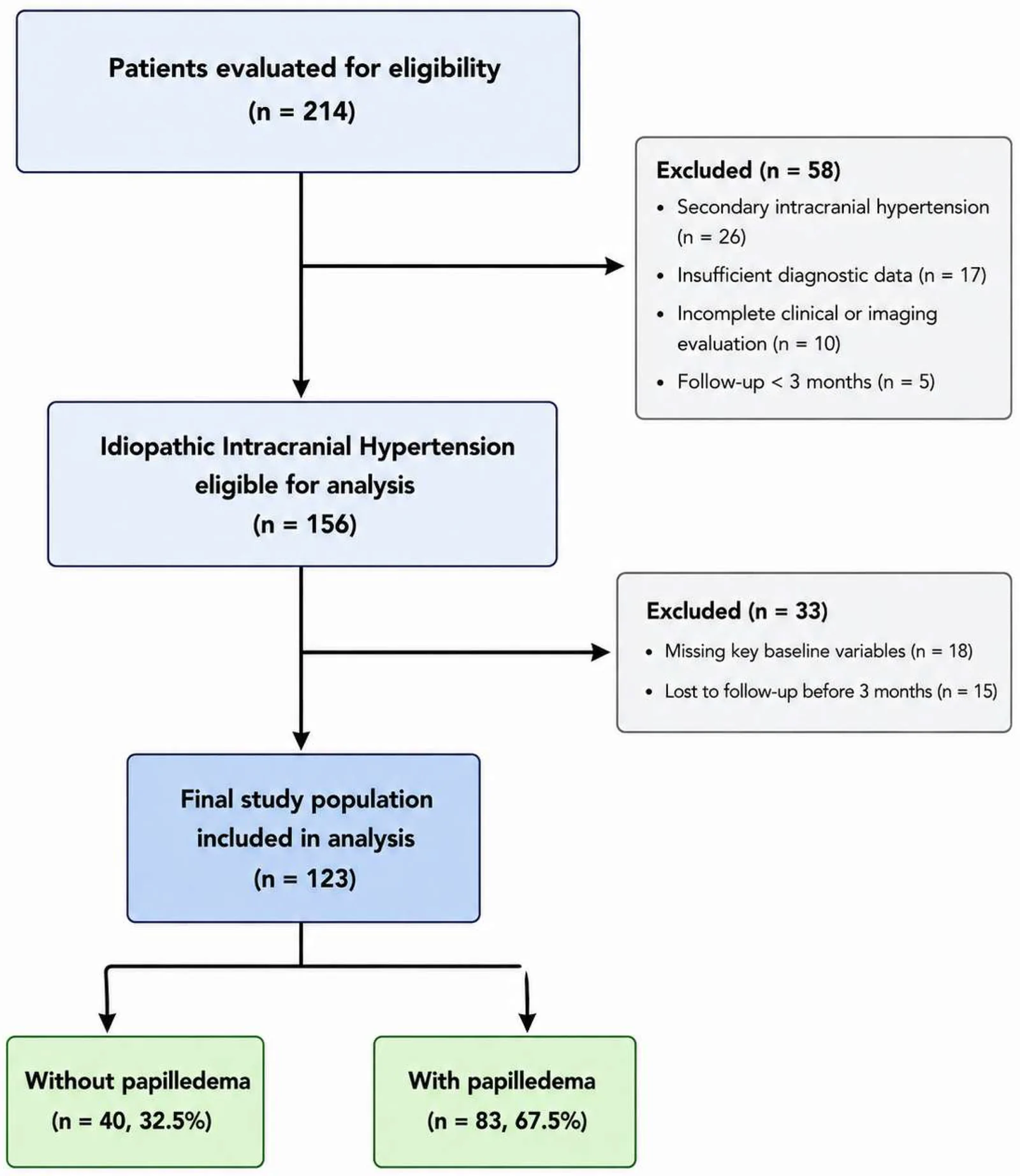
Flowchart of the study.

Of the 214 screened patients, 123 met the eligibility criteria and were included in the final analysis, comprising 83 patients with papilledema and 40 with IIHWOP (Figure 1).

### 2.2. Data Collection and Clinical Assessment

Clinical data were obtained from standardized medical records and the institutional IIH registry. Collected variables included demographic characteristics, comorbidities, headache features, associated symptoms, visual complaints, medication use, and documented aggravating or relieving factors.

All patients underwent comprehensive neurological and neuro-ophthalmologic evaluation, including visual acuity, color vision testing, ocular motility assessment, fundoscopy, and automated visual field testing. Papilledema status and visual field abnormalities were determined by neuro-ophthalmologic examination.

The impact of headache on daily life was extracted from routine clinical records and categorized as none, mild, moderate, or severe according to physician-documented assessments rather than a validated headache-specific questionnaire.

### 2.3. Neuroimaging and Lumbar Puncture

All patients underwent brain magnetic resonance imaging (MRI) and/or computed tomography (CT) to exclude secondary causes of intracranial hypertension. Imaging findings included empty sella, optic nerve flattening, optic nerve sheath enlargement, Meckel’s cave prominence, and MR venography abnormalities when available.

LP was performed according to routine clinical practice. Opening pressure, CSF findings, number of LP procedures, and post-LP headache occurrence were recorded.

### 2.4. Treatment and Outcome Assessment

Treatment was determined by treating physicians according to routine clinical practice and included acetazolamide, topiramate, analgesics, repeated LP, corticosteroids, optic nerve sheath fenestration, or CSF shunting when clinically indicated. Acetazolamide was generally initiated in patients with documented intracranial hypertension, particularly when papilledema, visual symptoms, or visual field abnormalities were present. Topiramate was considered primarily in patients with prominent headache or migraine-like symptoms, especially when weight reduction was also clinically desirable or when acetazolamide was not tolerated. However, during the study period, topiramate was not reimbursed for indications other than migraine under the national reimbursement system; therefore, access to this treatment was limited. Repeat LP was not used as a routine long-term treatment strategy but was performed in selected patients with persistent or worsening symptoms, diagnostic uncertainty, or a need for temporary pressure relief. Although optic nerve sheath fenestration and CSF diversion were among the available treatment options, no patient in the present cohort underwent these procedures during the analyzed follow-up period. CSF diversion procedures, including ventriculoperitoneal or lumboperitoneal shunting, were considered in patients with medically refractory intracranial hypertension, progressive visual dysfunction, or persistent disabling symptoms despite optimized conservative management. Treatment choice, dose adjustment, and escalation were individualized according to symptom burden, visual risk, treatment tolerance, comorbidities, and physician judgment.

Complete treatment response was defined as the resolution of symptoms or a clinically meaningful improvement in symptom burden, together with the absence of new or progressive visual complaints, stabilization or improvement of visual field findings, and no requirement for additional medical or surgical treatment during follow-up. Clinically meaningful symptom improvement was defined as a clear reduction in headache frequency, headache severity, or associated symptoms, as documented in routine follow-up records. Treatment response was assessed retrospectively from the clinical evaluations of the treating neurologist and neuro-ophthalmologist rather than by a standardized or validated outcome scale. Treatment persistence was defined as the interval between initiation of the primary treatment regimen and its discontinuation, switch, or escalation. Patients who remained on the initial regimen at their last follow-up were censored on that date. Because treatment decisions and outcome assessments were individualized and based on routine clinical practice, these measures should be interpreted as real-world clinical outcomes rather than standardized efficacy endpoints.

### 2.5. Statistical Analysis

Statistical analyses were performed using R version 4.5.2 (R Foundation for Statistical Computing, Vienna, Austria). Continuous variables were assessed using the Shapiro–Wilk test and are summarized as mean ± standard deviation or median (minimum–maximum), as appropriate. Categorical variables are presented as frequencies and percentages.

Group comparisons were performed using independent-sample *t*-tests or Wilcoxon rank-sum tests for continuous variables and chi-square or Fisher’s exact tests for categorical variables. Variables associated with complete treatment response in univariable analyses (*p* < 0.10), together with prespecified clinically relevant covariates, were considered for multivariable Firth logistic regression to reduce small-sample bias and address sparse data. To minimize overfitting, only variables demonstrating univariable association with treatment response or strong clinical relevance were retained. Results are reported as odds ratios (ORs) with 95% confidence intervals (CIs).

Treatment persistence was evaluated using Kaplan–Meier analysis and compared using the log-rank test. Because between-group comparisons were exploratory, no adjustment for multiple testing was applied, and secondary findings were considered hypothesis-generating. A two-sided *p*-value < 0.05 was considered statistically significant.

## 3. Results

A total of 214 patients were screened for eligibility, and 123 patients with IIH were included in the final analysis after applying exclusion criteria (Figure 1). Among them, 83 patients (67.5%) had papilledema, and 40 (32.5%) had IIHWOP. The overall median follow-up duration was 24 months (0–60 months).

Baseline demographic, clinical, neuro-ophthalmologic, imaging, and LP findings are summarized in Table 1. The mean age at diagnosis was 35.9 ± 10.2 years, and most patients were female (94.3%). The The mean body mass index (BMI) was 32.1 ± 5.1 kg/m^2^. Headaches were the predominant presenting symptom, affecting 96.8% of patients. Photophobia and phonophobia were reported by 73.2% and 74.0% of patients, respectively, while nausea/vomiting occurred in 30.1%. Papilledema was present in 67.5% of the cohort. Visual field abnormalities, visual field defects, peripheral constriction, and scotoma were observed in 51.2%, 53.7%, 83.7%, and 46.3% of patients, respectively. Optic nerve flattening was detected in 51.2% and abnormal MRI/CT findings in 23.6%. Median LP opening pressure was 33 cmH_2_O (26–80 cmH_2_O), and post-LP headache occurred in 52.0% of patients (Table 1). Detailed headache characteristics, pain profiles, aggravating and relieving factors, and neuro-ophthalmologic findings are presented in Supplementary Table S1.

Comparisons according to papilledema status are shown in Table 2. Among the 40 patients without papilledema, empty sella was present in 14 patients (35.0%), posterior globe or retinal flattening in 2 patients (5.0%), and documented optic nerve flattening in 27 patients (67.5%). MR venography was available in 39 of 40 patients (97.5%). Of these, 34 patients (87.2%) had a normal MR venogram, whereas three (7.7%) had venous sinus hypoplasia and two (5.1%) had venous sinus stenosis (These findings were derived from an additional descriptive analysis of the MR venography data. The relevant summary results are reported only narratively in the text). Follow-up duration was comparable between patients with and without papilledema (both median 24 months; range 0–60 months; *p* = 0.543). Age, sex distribution, and BMI were similar between groups. However, diabetes mellitus was more common among patients with IIHWOP than among those with papilledema (20.0% vs. 6.0%, *p* = 0.040).

Several headache-associated symptoms differed significantly between groups. Photophobia (87.5% vs. 66.3%, *p* = 0.023), phonophobia (87.5% vs. 67.5%, *p* = 0.031), and nausea/vomiting (50.0% vs. 20.5%, *p* = 0.002) were more frequent among IIHWOP. No significant differences were observed regarding headache duration, tinnitus, diplopia, eye pain, visual darkening, transient visual loss, permanent visual loss, or family history of headache (all *p* > 0.05).

The higher frequency of recorded optic nerve flattening (67.5% vs. 43.4%, *p* = 0.021) among patients classified as IIHWOP should not be interpreted as an independent phenotypic finding because optic nerve-related imaging findings contributed to the retrospective diagnostic classification. In the exploratory analysis, peripheral visual field constriction was more frequently observed in the IIHWOP group than in the papilledema group (95.0% vs. 78.3%, Fisher’s exact *p* = 0.019). Given the multiple unadjusted comparisons, this finding should be considered hypothesis-generating. Other visual field abnormalities, scotoma, enlarged blind spot, ocular motility disorders, and sixth nerve palsy did not differ significantly between groups (Table 2). Detailed comparisons of headache characteristics, pain localization, daily-life impact, aggravating and relieving factors, and detailed neuro-ophthalmologic subcategories also showed no significant between-group differences (Supplementary Table S2).

Imaging findings, including abnormal MRI/CT findings, empty sella, hyperintensity, and abnormal magnetic resonance venography (MRV) findings, were comparable between groups. Similarly, the number of LP procedures, LP opening pressure, and post-LP headache frequency did not differ significantly according to papilledema status (Table 2). Analgesic use was more frequent in the IIHWOP group (97.5% vs. 78.3%, *p* = 0.006). Acetazolamide use was recorded more frequently in patients with papilledema than in those without papilledema (10.8% vs. 0%, *p* = 0.030); however, this finding should be interpreted cautiously because treatment data reflected documented medication use in routine clinical records rather than a standardized treatment protocol.

**Figure 2:**
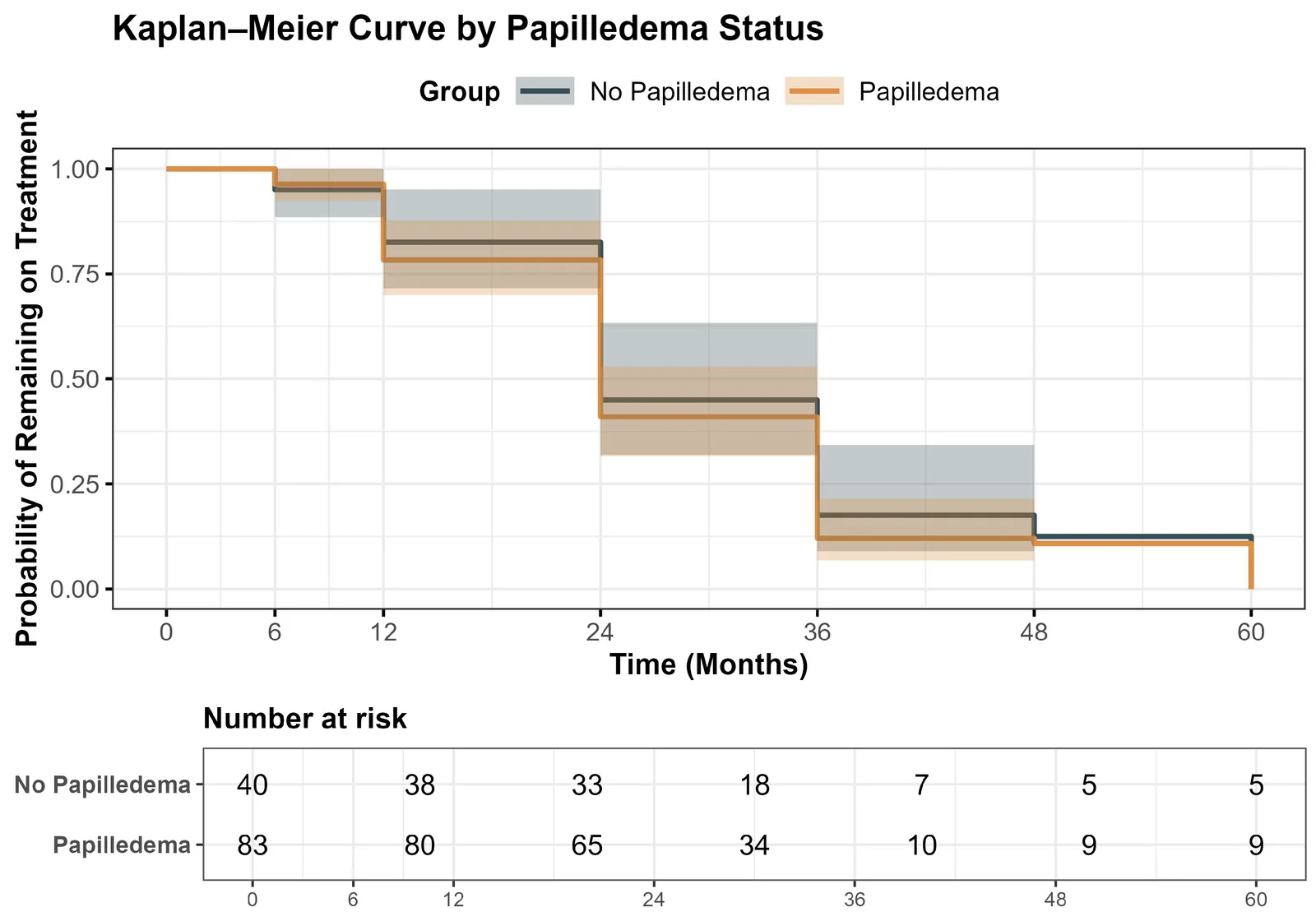
Kaplan–Meier curves for treatment persistence according to papilledema status. Shaded areas represent 95% confidence intervals. The numbers of patients at risk are presented below the graph. No significant between-group difference was observed using the log-rank test (χ^2^ = 0.269, *p* = 0.604).

Kaplan–Meier analysis showed no significant difference in treatment persistence between patients with and without papilledema (log-rank χ^2^ = 0.269, *p* = 0.604). Survival probabilities remained largely comparable throughout the follow-up intervals (6, 12, 24, 36, 48, and 60 months) (Figure 2).

Complete treatment response was available for all 123 patients. Complete treatment response occurred in 32 of 40 patients (80.0%) with IIHWOP and 63 of 83 patients (75.9%) with papilledema.

In univariable Firth logistic regression analyses (Table 3), papilledema was associated with higher odds of failure to achieve a complete treatment response (OR = 3.29, 95% CI: 1.02–11.24, *p* = 0.045), whereas higher LP opening pressure was associated with lower odds of treatment nonresponse (OR = 0.89, 95% CI: 0.84–0.94, *p* < 0.001). These associations remained significant in the multivariable model: papilledema was associated with higher odds of treatment nonresponse (OR = 4.38, 95% CI: 1.10–20.35, *p* = 0.036), whereas higher LP opening pressure remained associated with lower odds of treatment nonresponse (OR = 0.90, 95% CI: 0.84–0.95, *p* < 0.001). BMI and abnormal MRV findings were not independently associated with treatment nonresponse. The final model demonstrated a Nagelkerke R^2^ of 0.42. Given the limited number of nonresponse events and the resulting wide confidence intervals, these findings should be interpreted cautiously and require confirmation in larger cohorts.

## 4. Discussion

In this study, we compared the demographic, clinical, neuro-ophthalmologic, radiological, and treatment-related characteristics of patients with IIH with and without papilledema. Four principal findings emerged. First, nearly one-third of the cohort was retrospectively classified as having IIHWOP. Second, exploratory analyses suggested that photophobia, phonophobia, and nausea/vomiting were more frequent in patients without papilledema. Third, papilledema was independently associated with higher odds of failure to achieve a complete treatment response, whereas higher LP opening pressure was associated with lower odds of treatment nonresponse. Fourth, treatment persistence was comparable between the groups.

The prevalence of IIHWOP in our cohort (32.5%) was substantially higher than previously reported rates, which have ranged from approximately 5–6% in definite IIH cohorts to 10–15% in selected chronic headache populations, and therefore warrants careful interpretation. Digre et al. [16] reported a prevalence of approximately 5–6% among patients with definite IIH. More recently, Favoni et al. [9] reported IIHWOP frequencies ranging from 10% to 15% in selected chronic headache populations, whereas Emekli et al. suggested that papilledema-negative disease may be substantially underrecognized because current diagnostic frameworks rely heavily on papilledema. The higher prevalence observed in our study may partly reflect the tertiary referral nature of our neuro-ophthalmology and headache center, where patients with chronic refractory headache and suspected intracranial hypertension are frequently evaluated. In addition, patients without papilledema were classified as IIHWOP based on elevated LP opening pressure, normal CSF composition, exclusion of secondary causes, and supportive neuroimaging findings documented in routine clinical records. Nevertheless, because the retrospectively recorded imaging variables did not correspond uniformly to all four neuroimaging signs in the revised Friedman framework, some degree of diagnostic uncertainty and misclassification cannot be excluded. Our findings, together with previous reports, support the view that IIHWOP represents a clinically relevant presentation within the IIH spectrum. However, several between-group differences identified in this study were exploratory and should be interpreted cautiously until confirmed in independent cohorts [12]. This interpretation is further supported by a recent systematic review by Labella Alvarez et al. [18], which emphasized that IIHWOP should be regarded as a distinct and underrecognized phenotype within the IIH spectrum and highlighted ongoing diagnostic uncertainties and unmet needs regarding long-term management strategies. Although interobserver differences in papilledema recognition cannot be completely excluded, all patients underwent formal neuro-ophthalmologic evaluation.

In exploratory unadjusted analyses, photophobia, phonophobia, and nausea/vomiting were more frequent among patients with IIHWOP than among those with papilledema. In our cohort, photophobia was present in 87.5% versus 66.3%, phonophobia in 87.5% versus 67.5%, and nausea/vomiting in 50.0% versus 20.5% of patients with IIHWOP and papilledema, respectively. These symptoms are characteristic of migraine-like headache phenotypes and suggest that symptom burden may not be directly related to the presence of papilledema [1]. Previous studies have highlighted the overlap between chronic headache disorders and IIHWOP, particularly among patients presenting with migraine-like headaches [9,10,11]. Favoni et al. [9] demonstrated that IIHWOP may be underrecognized among patients with chronic refractory headache, while Malky et al. further emphasized its occurrence in chronic migraine populations. Similarly, Beri et al. [8] reported that patients with IIHWOP frequently present with chronic daily headaches exhibiting migraine-like characteristics. Our findings are consistent with previous reports suggesting that migraine-like symptoms may be more common in patients with IIHWOP. Because these comparisons were exploratory and were not adjusted for multiple testing, they should be considered hypothesis-generating. Interestingly, the prevalence of a previous migraine diagnosis was comparable between groups (42.5% vs. 36.1%, *p* = 0.813), despite the markedly higher frequency of migraine-associated symptoms among patients with IIHWOP. This discrepancy may reflect differences in symptom expression among patients with IIHWOP; however, this interpretation remains speculative and requires confirmation in prospective studies. Consequently, IIHWOP may initially be classified within the spectrum of primary headache disorders, potentially contributing to delayed recognition or underdiagnosis.

The absence of significant differences in LP opening pressure between groups is consistent with previous evidence indicating that papilledema development is not determined solely by the magnitude of intracranial pressure. Patients with similar CSF opening pressures may exhibit markedly different neuro-ophthalmologic manifestations, suggesting that anatomical and physiological factors influence pressure transmission to the optic nerve head [10,11,16,19]. Digre et al. [16] similarly reported comparable opening pressures in patients with and without papilledema. Variations in optic nerve sheath anatomy, lamina cribrosa compliance, optic canal dimensions, and translaminar pressure gradients have all been proposed as potential explanations.

Our neuro-ophthalmologic findings further support this concept. Optic nerve flattening was more frequently observed in the IIHWOP group (67.5% vs. 43.4%, *p* = 0.021). However, because optic nerve flattening contributed to the diagnostic classification of IIHWOP in the present study, this finding should be interpreted as supportive of the applied diagnostic framework rather than as an independent phenotypic difference between groups. In contrast, visual field abnormalities, scotoma, enlarged blind spots, and sixth nerve palsy were comparable between the two groups, indicating broadly similar functional neuro-ophthalmologic findings despite the absence of papilledema. These observations are consistent with previous studies highlighting the value of neuroimaging in patients with suspected IIHWOP. Beier et al. [13] identified posterior globe flattening, optic nerve protrusion, and transverse sinus stenosis as among the most informative MRI markers of IIH. Likewise, radiological signs of intracranial hypertension may be present even in symptomatic patients whose opening pressures do not exceed traditional diagnostic thresholds [14]. Taken together, these observations support the complementary role of neuroimaging in the evaluation of patients with suspected IIHWOP when papilledema is absent, although the imaging findings should be interpreted within the context of the diagnostic framework used in this study. Recent device-focused evidence and experimental models have renewed interest in venous sinus pathology as both a mechanistic contributor and a potential therapeutic target in selected patients [5].

Another important observation was the lack of significant differences in visual field abnormalities between the groups. Although papilledema has traditionally been considered the principal mechanism responsible for visual dysfunction in IIH [1,16], accumulating evidence indicates that visual symptoms and visual field abnormalities may occur even without clinically visible optic disc edema [10,11,20]. Our findings are consistent with previous studies and support comprehensive neuro-ophthalmologic assessment regardless of papilledema status.

The relationship between papilledema and treatment outcomes remains incompletely understood. In our multivariable analysis, papilledema was independently associated with higher odds of failure to achieve a complete treatment response. In this study, complete response was defined as an overall composite clinical outcome comprising symptom improvement, stabilization of visual complaints and visual field findings, and the absence of treatment escalation. However, this association should be interpreted cautiously because treatment response was assessed retrospectively from routine clinical records, was not based on a validated outcome scale, and combined subjective symptom improvement with ophthalmologic and treatment-related components. In addition, treatment selection and follow-up assessments were not standardized, and residual confounding related to disease severity, visual risk, physician preference, and treatment intensity cannot be excluded. One possible explanation is that papilledema may identify a phenotype with greater ophthalmologic involvement or disease burden, potentially reducing the likelihood of achieving a complete clinical response. However, this interpretation remains speculative because treatment selection, monitoring intensity, and outcome assessment were not standardized.

Treatment persistence did not differ significantly between the groups. Because treatment was not standardized and reflected routine clinical practice, medication use was influenced by symptom burden, visual risk, treatment tolerability, and physician preference. Therefore, treatment patterns should not be interpreted as evidence of differential treatment efficacy. Despite differences in symptom profiles, patients with and without papilledema may experience similar long-term treatment continuation patterns [21]. The relatively low acetazolamide use reflected real-world retrospective practice patterns across the study period rather than a standardized protocol. These findings suggest that treatment continuation patterns may be broadly similar across phenotypes. Nevertheless, the absence of papilledema should not be assumed to indicate a benign clinical course.

Recent advances in imaging further support a broader conceptualization of IIH. Optical coherence tomography (OCT) has emerged as a sensitive and objective tool for monitoring disease activity, with evidence suggesting that OCT-derived structural biomarkers may outperform both funduscopic examination and intracranial pressure measurements in longitudinal assessment [20]. OCT parameters correlate with papilledema severity [20,22], and a recent systematic review demonstrated significant associations between CSF pressure and OCT-derived structural measures [23]. These findings support the potential role of OCT as a quantitative biomarker for disease monitoring. However, OCT was not routinely performed throughout the entire study period at our institution because of the retrospective design and evolving clinical practice patterns. Therefore, OCT data were not consistently available for all patients and could not be systematically incorporated into the present analyses.

Several limitations should be acknowledged. First, OCT data were not consistently available, preventing objective structural assessment of the optic nerve head and retinal nerve fiber layer. The absence of OCT measurements limits the interpretation of visual pathway involvement, particularly in patients without visible papilledema. Second, the retrospective, single-center design conducted at a tertiary referral center may have introduced selection bias by enriching the cohort with more complex or diagnostically challenging cases, thereby limiting the generalizability of the findings. Third, treatment selection depended on physician judgment, symptom severity, visual risk, patient tolerance, and evolving institutional practice, which may have influenced treatment-related outcomes. In addition, limited reimbursement for topiramate outside the indication of migraine may have restricted treatment access and introduced socioeconomic variability into medication selection. Treatment response was based on clinician assessment rather than a validated outcome measure; outcome assessment was not blinded, patient-reported outcomes were unavailable, and retrospective documentation may have introduced misclassification. In addition, because IIHWOP remains an evolving and sometimes controversial entity, diagnostic misclassification cannot be completely excluded. Moreover, the neuroimaging variables available in the retrospective records did not correspond uniformly to all four neuroimaging signs specified in the revised Friedman framework. In particular, the recorded findings of optic nerve flattening and nonspecific MR venographic abnormalities could not be considered equivalent to perioptic subarachnoid space distension or transverse venous sinus stenosis. Therefore, these findings should be interpreted as descriptive imaging characteristics rather than evidence of complete fulfillment of the neuroimaging component of the revised Friedman criteria. This limitation introduces diagnostic uncertainty and underscores the need for patient-level re-evaluation of the original imaging studies. Finally, longitudinal changes in neuroimaging findings and visual function were not systematically evaluated.

Despite these limitations, our study represents one of the larger comparative analyses of patients with IIH with and without papilledema and provides a comprehensive evaluation of clinical, neuro-ophthalmologic, radiological, and treatment-related characteristics. The findings contribute to the growing body of evidence suggesting that IIHWOP represents a clinically relevant presentation within the IIH spectrum.

## 5. Conclusions

Exploratory analyses suggested that patients retrospectively classified as having IIHWOP may experience more prominent migraine-like symptoms. The more frequent documentation of optic nerve flattening in this group should be interpreted cautiously because neuroimaging findings contributed to the retrospective diagnostic classification. Within this retrospective cohort, papilledema was independently associated with higher odds of failure to achieve a complete treatment response, whereas higher LP opening pressure was associated with lower odds of treatment nonresponse. Given the retrospective assessment of treatment response and the limited number of nonresponse events, these associations should be interpreted cautiously and should not be considered causal. The observed between-group differences require confirmation in larger prospective studies using standardized diagnostic, imaging, and outcome criteria.

## Figures and Tables

**Table 1:** Baseline demographic, clinical, and neuro-ophthalmologic characteristics of the study population (*n* = 123).

Variable	Total (*n* = 123)	Variable	Total (*n* = 123)
**Demographic Characteristics**		Family history of headaches	32 (26.0)
Age at diagnosis (years) *	35.9 ± 10.2	**Neuro-ophthalmologic Findings**	
Female sex	116 (94.3)	Papilledema present	83 (67.5)
Height (cm) *	159.0 ± 6.7	Ocular motility disorder present	5 (4.1)
Weight (kg) *	81 (57–128)	Sixth nerve palsy present	2 (1.6)
Body mass index (kg/m^2^) *	32.1 ± 5.1	Color vision impairment present	28 (22.8)
Presence of any comorbidity	24 (19.5)	Visual field abnormality present	63 (51.2)
*Hypertension*	16 (13.0)	Scotoma present	57 (46.3)
*Diabetes mellitus*	13 (10.6)	Enlarged blind spot present	20 (16.3)
**Core Clinical Features**		Peripheral constriction present	103 (83.7)
Headache	119 (96.8)	Visual field defect present	66 (53.7)
Headache duration (hours) *	7 (0–120)	**Imaging Findings**	
Current pain severity score *	4 (0–10)	Abnormal MRI/CT findings	29 (23.6)
Presence of nausea/vomiting	37 (30.1)	Empty sella	33 (26.8)
Photophobia	90 (73.2)	Hyperintensity	13 (10.6)
Phonophobia	91 (74.0)	Optic nerve flattening	63 (51.2)
Tinnitus	24 (19.5)	MR venography performed	122 (99.2)
Diplopia	18 (14.6)	Abnormal MRV findings	17 (13.9)
Eye pain	62 (50.4)	**Lumbar Puncture Findings**	
Visual darkening	33 (26.8)	Number of lumbar punctures *	1 (1–3)
Transient visual loss	29 (23.6)	LP opening pressure (cmH_2_O) *	33 (26–80)
Permanent visual loss	5 (4.1)	Post-lumbar puncture headache	64 (52.0)
Median follow-up duration (months) *	24 (0–60)		

**Notes:** * Continuous variables are presented as the mean ± standard deviation or median (minimum–maximum), as appropriate; categorical variables are presented as *n* (%). **Abbreviations:** LP, lumbar puncture; MRI/CT, magnetic resonance imaging/computed tomography; MRV, magnetic resonance venography.

**Table 2:** Comparison of demographic, clinical, neuro-ophthalmologic, imaging, and treatment characteristics according to papilledema status in patients with idiopathic intracranial hypertension.

Variable	IIH Without Papilledema, *n* (%) *n* = 40	IIH with Papilledema, *n* (%) *n* = 83	*p*
**Demographic Characteristics**			
Age at diagnosis (years) *	37.5 ± 9.8	35.3 ± 10.3	0.235
Female sex	39 (97.5)	77 (92.8)	0.426
Body mass index (kg/m^2^) *	32.3 ± 4.1	32.1 ± 5.6	0.873
Diabetes mellitus	8 (20.0)	5 (6.0)	**0.040**
**Core Clinical Features**			
Headache	40 (100.0)	79 (95.2)	0.303
Headache duration (hours) *	7.5 (1–120)	6 (0–120)	0.106
Photophobia	35 (87.5)	55 (66.3)	**0.023**
Phonophobia	35 (87.5)	56 (67.5)	**0.031**
Nausea/Vomiting	20 (50.0)	17 (20.5)	**0.002**
Tinnitus	5 (12.5)	19 (22.9)	0.263
Diplopia	3 (7.5)	15 (18.1)	0.174
Eye pain	24 (60.0)	38 (45.8)	0.199
Visual darkening	7 (17.5)	26 (31.3)	0.160
Transient visual loss	7 (17.5)	22 (26.5)	0.381
Permanent visual loss	1 (2.5)	4 (4.8)	>0.999
Family history of headache	14 (35.0)	18 (21.7)	0.175
**Neuro-ophthalmologic Findings**			
Color vision impairment	5 (12.5)	23 (27.7)	0.356
Visual field abnormality	20 (50.0)	43 (51.8)	>0.999
Scotoma	21 (52.5)	36 (43.4)	0.440
Enlarged blind spot	10 (25.0)	10 (12.0)	0.115
Peripheral constriction	38 (95.0)	65 (78.3)	**0.019**
Visual field defect	17 (42.5)	49 (59.0)	0.122
Optic nerve flattening	27 (67.5)	36 (43.4)	0.021
Ocular motility disorder	1 (2.5)	4 (4.8)	>0.999
Sixth nerve palsy	0 (0.0)	2 (2.4)	>0.999
**Imaging Findings**			
Abnormal MRI/CT findings	8 (20.0)	21 (25.3)	0.673
Empty sella	14 (35.0)	19 (22.9)	0.229
Hyperintensity	6 (15.0)	7 (8.4)	0.426
Abnormal MRV findings	5 (12.8)	12 (14.5)	0.170
**Lumbar Puncture Findings**			
Number of LPs *	1 (1–2)	1 (1–3)	0.538
LP opening pressure (cmH_2_O) *	32 (26–65)	33 (26–80)	0.463
Post-LP headache	23 (57.5)	41 (49.4)	0.516
**Treatment**			
Analgesic use	39 (97.5)	65 (78.3)	**0.006**
Acetazolamide use	0 (0.0)	9 (10.8)	**0.030**
Median follow-up duration (months) *	24 (0–60)	24 (0–60)	0.543

**Notes:** * Continuous variables are presented as the mean ± standard deviation or median (minimum–maximum), as appropriate; categorical variables are presented as *n* (%). Continuous variables were compared using the Wilcoxon rank-sum test. Categorical variables were compared using Pearson’s chi-square test with Yates’ continuity correction or Fisher’s exact test, as appropriate. All between-group comparisons were exploratory and were not adjusted for multiple testing. The bold format indicates statistically significant *p*-values (*p* < 0.05). **Abbreviations:** IIH, idiopathic intracranial hypertension; LP, lumbar puncture; MRI/CT, magnetic resonance imaging/computed tomography; MRV, magnetic resonance venography.

**Table 3:** Clinical factors associated with failure to achieve a complete treatment response.

	Univariable		Multivariable	
	OR (CI)	*p*	OR (CI)	*p*
Papilledema Status	3.29 (1.02–11.24)	**0.045**	4.38 (1.10–20.35)	**0.036**
LP pressure	0.89 (0.84–0.94)	**<0.001**	0.90 (0.84–0.95)	**<0.001**
BMI	0.91 (0.82–1.02)	0.117	0.87 (0.72–1.03)	0.114
Abnormal MRV findings	1.34 (0.29–12.92)	0.741	1.41 (0.25–15.03)	0.721

Nagelkerke R^2^: 0.42. The bold format indicates statistically significant *p*-values (*p* < 0.05). **Abbreviations:** CI, confidence interval; LP, lumbar puncture; MRV, magnetic resonance venography; OR, odds ratio.

## Data Availability

The datasets generated and/or analyzed during the current study are not publicly available due to privacy and ethical restrictions but are available from the corresponding author upon reasonable request.

## Supplementary Materials

The following supporting information can be downloaded at https://www.mdpi.com/article/10.3390/brainsci16080798/s1, Supplementary Table S1. Detailed symptom characteristics, pain profile, and neuro-ophthalmologic findings of the study population (*n* = 123). Supplementary Table S2. Detailed comparison of clinical symptoms, pain characteristics, and neuro-ophthalmologic findings according to papilledema status.

## Abbreviations

The following abbreviations are used in this manuscript:

BMI	Body Mass Index
CI	Confidence Interval
CSF	Cerebrospinal Fluid
CT	Computed Tomography
ICP	Intracranial Pressure
IIH	Idiopathic Intracranial Hypertension
IIHWOP	Idiopathic Intracranial Hypertension Without Papilledema
LP	Lumbar Puncture
MRI	Magnetic Resonance Imaging
MRV	Magnetic Resonance Venography
OCT	Optical Coherence Tomography
OR	Odds Ratio
