# Supplementary material for: Idiopathic Intracranial Hypertension with and Without Papilledema: A Comparative Cohort Study
Source: Brain Sci. 2026 Jul 28;16(8):798. doi: 10.3390/brainsci16080798 (PMC13511475; doi:10.3390/brainsci16080798)
Supplement: Supplementary file 1 [file brainsci-16-00798-s001.zip › brainsci-4431777-supplementary.pdf]

Supplementary Table S1. Detailed Symptom Characteristics, Pain Profile, and Neuro-ophthalmologic Findings of the Study Population (n=123).

Variable	Total (n=123)	Variable	Total (n=123)
**Pain Characteristics**		**Pain-Relieving Factors†**	
Throbbing pain	72 (59.0)	*Presence of any relieving factor*	113 (91.9)
Dull/pressing pain	35 (28.7)	*Rest*	84 (68.3)
Stabbing/piercing pain	13 (10.7)	*Sleep*	82 (66.7)
Other pain characteristics	2 (1.6)	*Medication use*	54 (43.9)
**Headache episode duration categories**		*Exercise*	3 (2.4)
<1 hour	7 (5.7)	*Other relieving factors*	4 (3.3)
1–3 hours	21 (17.2)	**Pain-Aggravating Factors†**	
3–12 hours	19 (15.6)	*Presence of any aggravating factor*	109 (88.6)
12–24 hours	44 (36.1)	*Stress*	95 (77.2)
1–3 days	28 (23.0)	*Sleep deprivation*	55 (44.7)
>3 days	3 (2.5)	*Climate/weather changes*	47 (38.2)
Unilateral pain	73 (59.4)	*Posture*	40 (32.5)
Bilateral pain	53 (43.1)	*Physical activity*	37 (30.1)
Multiple pain locations	81 (69.8)	*Menstruation*	25 (20.3)
**Pain Location**		*Coughing/straining*	15 (12.2)
Periorbital	46 (37.7)	*Alcohol*	6 (4.9)
Frontal	37 (30.3)	*Other aggravating factors*	1 (0.8)
Parietal	19 (15.6)	**Detailed Neuro-ophthalmologic Findings**	
Temporal	11 (9.0)	*Right ocular motility disorder*	1 (0.8)
Occipital	4 (3.3)	*Left ocular motility disorder*	1 (0.8)
Nuchal	2 (1.6)	*Bilateral ocular motility disorder*	3 (2.4)
Other	3 (2.5)	*Left sixth nerve palsy*	1 (0.8)
**Time of Day**		*Bilateral sixth nerve palsy*	1 (0.8)
Morning	47 (38.2)	*Right color vision impairment*	7 (5.7)
Afternoon	34 (27.6)	*Left color vision impairment*	2 (1.6)
Night	13 (10.6)	*Bilateral color vision impairment*	19 (15.5)
No specific pattern	29 (23.6)	*Right visual field abnormality*	6 (4.9)
**Associated Symptoms**		*Left visual field abnormality*	5 (4.1)
Nausea	71 (57.7)	*Bilateral visual field abnormality*	52 (42.2)
Vomiting	28 (22.8)	*Right scotoma*	6 (4.9)
Dizziness	14 (11.4)	*Left scotoma*	3 (2.4)
Falls	4 (3.3)	*Bilateral scotoma*	48 (39.0)
**Impact on Daily Life**		*Right enlarged blind spot*	4 (3.3)
None	10 (8.1)	*Left enlarged blind spot*	2 (1.6)
Mild	19 (15.5)	*Bilateral enlarged blind spot*	14 (11.4)
Moderate	48 (39.0)	*Right peripheral constriction*	11 (8.9)
Severe	46 (37.4)	*Left peripheral constriction*	4 (3.3)
**Previous Headache Diagnoses**		*Bilateral peripheral constriction*	88 (71.5)
Migraine	47 (38.2)	*Right visual field defect*	9 (7.3)
Tension-type headache	12 (9.8)	*Left visual field defect*	5 (4.1)

Other diagnoses	64 (52.0)	*Bilateral visual field defect*	52 (42.3)

Notes: † Multiple responses were allowed; therefore, percentages may exceed 100%. Headache episode duration categories were available for 122 patients and were analyzed separately from the continuously recorded headache duration in hours. Nausea/vomiting denotes the combined presenting-symptom variable recorded in the clinical history, whereas nausea and vomiting were also recorded separately as headache-associated symptoms.

Supplementary Table S2. Detailed Comparison of Clinical Symptoms, Pain Characteristics, and Neuro-ophthalmologic Findings According to Papilledema Status.

Variable	IIH without Papilledema, n (%) n=40	IIH with Papilledema, n (%) n=83	p
**Pain Characteristics**			0.567
Throbbing	27 (67.5)	45 (54.9)	
Dull/pressing	10 (25.0)	25 (30.5)	
Stabbing/piercing	3 (7.5)	10 (12.2)	
Other	0 (0.0)	2 (2.4)	
**Pain Duration**			0.240
<1 hour	1 (2.5)	6 (7.3)	
1–3 hours	4 (10.0)	17 (20.7)	
3–12 hours	8 (20.0)	11 (13.4)	
12–24 hours	14 (35.0)	30 (36.6)	
1–3 days	13 (32.5)	15 (18.3)	
>3 days	0 (0.0)	3 (3.7)	
**Unilateral pain**	23 (57.5)	50 (60.2)	0.925
**Bilateral pain**	19 (47.5)	34 (41.0)	0.623
**Multiple pain locations**	25 (62.5)	56 (73.7)	0.301
**Pain Location**			0.387
Periorbital	14 (35.0)	32 (39.0)	
Frontal	14 (35.0)	23 (28.1)	
Parietal	6 (15.0)	13 (15.9)	
Temporal	2 (5.0)	9 (11.0)	
Occipital	3 (7.5)	1 (1.2)	
Nuchal	1 (2.5)	1 (1.2)	
Other	0 (0.0)	3 (3.6)	
**Time of Day**			0.081
Morning	21 (52.5)	26 (31.3)	
Afternoon	6 (15.0)	28 (33.7)	
Night	4 (10.0)	9 (10.8)	
No specific pattern	9 (22.5)	20 (24.1)	
**Associated Symptoms**			
Dizziness	4 (10.0)	10 (12.1)	>0.999
Falls	0 (0.0)	4 (4.8)	0.303
**Pain-Relieving Factors†**	38 (95.0)	75 (90.4)	0.497
Rest	32 (80.0)	52 (62.7)	0.084
Sleep	31 (77.5)	51 (61.5)	0.118
Medication use	17 (42.5)	37 (44.6)	0.981
Exercise	2 (5.0)	1 (1.2)	0.247
Other relieving factors	3 (7.5)	1 (1.2)	0.100
**Pain-Aggravating Factors†**	36 (90.0)	73 (88.0)	>0.999
Stress	31 (77.5)	64 (77.1)	>0.999
Sleep deprivation	17 (42.5)	38 (45.8)	0.881
Climate/weather	20 (50.0)	27 (32.5)	0.095
Posture	11 (27.5)	29 (34.9)	0.535
Physical activity	9 (22.5)	28 (33.7)	0.288

Menstruation	10 (25.0)	15 (18.1)	0.512
Coughing/straining	3 (7.5)	12 (14.5)	0.381
Alcohol	3 (7.5)	3 (3.6)	0.389
Other aggravating factors	1 (2.5)	0 (0.0)	0.325
**Impact on Daily Life**			0.389
None	1 (2.5)	9 (10.8)	
Mild	5 (12.5)	14 (16.9)	
Moderate	17 (42.5)	31 (37.4)	
Severe	17 (42.5)	29 (34.9)	
**Previous Headache Diagnoses**			0.813
Migraine	17 (42.5)	30 (36.1)	
Tension-type headache	3 (7.5)	9 (10.8)	
Other	20 (50.0)	44 (53.0)	
**Detailed Neuro-ophthalmologic Findings**			
**Ocular motility disorder**			
Right ocular motility disorder	0 (0.0)	1 (1.2)	>0.999
Left ocular motility disorder	0 (0.0)	1 (1.2)	
Bilateral ocular motility disorder	1 (2.5)	2 (2.4)	
**Sixth nerve palsy**			>0.999
Left sixth nerve palsy	0 (0.0)	1 (1.2)	
Bilateral sixth nerve palsy	0 (0.0)	1 (1.2)	
**Color vision impairment**			0.356
Right color vision impairment	1 (2.5)	6 (7.2)	
Left color vision impairment	0 (0.0)	2 (2.4)	
Bilateral color vision impairment	4 (10.0)	15 (18.1)	
**Visual field findings**			0.230
Right visual field finding	4 (10.0)	2 (2.4)	
Left visual field finding	2 (5.0)	3 (3.6)	
Bilateral visual field finding	14 (35.0)	38 (45.8)	
**Scotoma**			0.672
Right scotoma	3 (7.5)	3 (3.6)	
Left scotoma	1 (2.5)	2 (2.4)	
Bilateral scotoma	17 (42.5)	31 (37.4)	
**Enlarged blind spot**			0.234
Right enlarged blind spot	2 (5.0)	2 (2.4)	
Left enlarged blind spot	1 (2.5)	1 (1.2)	
Bilateral enlarged blind spot	7 (17.5)	7 (8.4)	
**Peripheral constriction**			0.076
Right peripheral constriction	5 (12.5)	6 (7.2)	
Left peripheral constriction	1 (2.5)	3 (3.6)	
Bilateral peripheral constriction	32 (80.0)	56 (67.5)	
**Visual field defect**			0.204
Right visual field defect	4 (10.0)	5 (6.0)	
Left visual field defect	1 (2.5)	4 (4.8)	
Bilateral visual field defect	12 (30.0)	40 (48.2)	
**Retinal flattening**	2 (5.0)	5 (6.0)	>0.999
